# Benzene Exposure and Biomarkers in Alveolar Air and Urine Among Deck Crews on Tankers Transporting Gasoline

**DOI:** 10.1093/annweh/wxz055

**Published:** 2019-08-06

**Authors:** Karl Forsell, Ingrid Liljelind, Göran Ljungkvist, Rolf Nordlinder, Eva Andersson, Ralph Nilsson

**Affiliations:** 1 Occupational and Environmental Medicine, Sahlgrenska Academy, Gothenburg University, Göteborg, Sweden; 2 Occupational and Environmental Medicine, Umeå University, Umeå, Sweden

**Keywords:** biological monitoring, chemical/product tanker, gasoline, seafarer, *t*,*t*-muconic acid

## Abstract

**Introduction:**

Increased rates of leukaemia have been found among tanker crews. Occupational exposures to the leukomogen benzene during loading, unloading, and tank cleaning are possible causes. Studies on older types of tankers carrying gasoline with most handling being done manually have revealed important exposures to benzene. Our study explores benzene exposures on tankers with both automatic and manual systems. Correlations between benzene exposure and benzene in alveolar air (AlvBe), benzene in urine (UBe), and *trans*,*trans*-muconic acid (ttMA) in urine were investigated.

**Methods:**

Forty-three male seafarers (22 deck crewmembers and 21 not on deck) on five Swedish different product and chemical tankers transporting 95- or 98-octane gasoline were investigated between 1995 and 1998. The tankers used closed systems for the loading and unloading of gasoline but stripping and tank cleaning were done manually. Benzene in respiratory air was measured using personal passive dosimeters during a 4-h work shift. Samples for biomarker analyses were collected pre- and post-shift. Smoking did occur and crewmembers did not use any respiratory protection during work.

**Results:**

The average 4-h benzene exposure level for exposed was 0.45 mg m^−3^ and for non-exposed 0.02 mg m^−3^. Benzene exposure varied with type of work (range 0.02–143 mg m^−3^). AlvBe, UBe, and ttMA were significantly higher in post-shift samples among exposed and correlated with exposure level (*r* = 0.89, 0.74, and 0.57, respectively). Smoking did not change the level of significance among exposed.

**Discussion:**

Benzene in alveolar air, unmetabolized benzene, and ttMA in urine are potential biomarkers for occupational benzene exposure. Biomarkers were detectable in non-exposed, suggesting benzene exposure even for other work categories on board tankers. Work on tankers carrying gasoline with more or less closed handling of the cargo may still lead to significant benzene exposure for deck crewmembers, and even exceed the Swedish Occupational Exposure Limit (OEL; 8-h time-weighted average [TWA]) of 1.5 mg m^−3^.

## Introduction

Benzene is a well-established risk factor for haematologic malignancy (e.g. leukaemia), as well as depression of the haematological and immunological systems [International Agency for Research on Cancer (IARC), 2012; European Chemicals Agency (ECHA), 2017]. Increased risks for haematologic malignancy for tanker crews have been reported in some studies on older types of tankers (Nilsson *et al.*, 1998; Saarni *et al.*, 2002).

Tanker deck crewmembers may be exposed to benzene in air during loading and unloading and maintenance work, such as tank cleaning operations. Benzene exposure may vary with the benzene content of the cargo, venting mechanisms and protective equipment used, and ambient meteorological factors (Mowe *et al.*, 1977; Moen *et al.*, 1988; Kirkeleit *et al.*, 2006). There is certainly a difference in benzene exposure levels between modern shipping and older types of tankers. Several work tasks switched during 1990s in Western countries to semi-automatic or completely automatic manoeuvres from being manually performed. Manual work tasks were typically sounding the tanks, visual inspections for topping (the last part of loading) and stripping (the last part of unloading), and tank cleaning, which was done by a worker physically present inside the tanks, removing excess petroleum products with a water jet or a similar device. Furthermore, the European allowance limit for benzene in gasoline dropped from a maximum of 5% v/v to 1% v/v in 1998 [European Union (EU), 1998], possibly contributing to reduced occupational benzene exposures on tankers.

Inhalation of benzene is usually the most important exposure route in occupational settings with an absorption ratio of 45–90%, depending on dose and pulmonary ventilation rate (Pekari *et al.*, 1992; Boogaard and van Sittert, 1996; Arnold *et al.*, 2013). Although skin exposure may be present, transdermal absorption of benzene is generally considered much less important (ECHA, 2017).

Elimination of benzene involves both that of the unmetabolized benzene and elimination by complex metabolic pathways that vary in activity with exposure dose (Scherer *et al.*, 1998; ECHA, 2017). Of the total benzene dose absorbed, 17% is eliminated unchanged in breath. In urine, 2–25% of absorbed benzene is excreted as *trans*,*trans-*muconic acid (ttMA), 0.1% as unmetabolized benzene, and <1% as *S*-phenylmercapturic acid (SPMA). Other important, but less specific, benzene metabolites are phenol, hydroquinone, and catechol (Boogaard and van Sittert, 1995; Ghittori *et al.*, 1995; Ong *et al.*, 1996; Scherer *et al.*, 1998; Arnold *et al.*, 2013). Suggested biomarkers for benzene exposure in occupational settings are urinary samples of unmetabolized benzene or SPMA [Arnold *et al.*, 2013; ECHA, 2017; Committee for Risk Assessment – ECHA (RAC–ECHA), 2018].

The Swedish Occupational Exposure Limit (OEL) for benzene exposure dates from 1990 and equals 1.5 mg m^−3^, with a Short-Term Exposure Limit (STEL), equivalent to 15-min average exposure, of 9 mg m^−3^ (Swedish Work Environment Authority, 2018). The International Maritime Organization (IMO) recommends an OEL of 1 ppm, equivalent to 3.25 mg m^−3^, and a STEL of 5 ppm, equivalent to 16 mg m^−3^ (IMO, 2003). The OEL for IMO member states is the same as that for the EU.

The EU is currently, however, preparing for a much lower OEL. In 2017, the ECHA suggested a new OEL of 0.1 ppm, or 0.3 mg m^−3^, which, assuming a linear risk assessment approach, would correspond to four extra cases of leukaemia in 10 000 exposed workers (ECHA, 2017). However, a year later (2018), the ECHA RAC proposed an even lower OEL of 0.05 ppm, or 0.16 mg m^−3^ (RAC, 2018). This OEL should avoid chromosomal changes in benzene-exposed workers and entail a ‘no significant residual cancer risk’. RAC proposed the use of either (unmetabolized) benzene or SPMA for exposure assessment in relation to so-called Biological Limit Values (BLV) (0.7 µg L^−1^ and 2 µg g^−1^ creatinine, respectively), both analysed in post-shift or post-exposure urinary samples. Knowledge of benzene exposure levels and associations with biomarkers during work on tankers carrying petroleum products is scarce, especially for tankers after the introduction of closed cargo handling. Furthermore, there is little knowledge of how biomarkers sampled from tanker crewmembers would agree with their benzene exposure levels, that is, if biomarkers can be used for exposure assessment for this type of work.

The overall aim with the study was to study benzene exposure and associated biomarker levels during field conditions for work on tankers carrying gasoline with both closed and manual operations. The objectives were to increase the scientific knowledge on benzene exposures for deck workers on tankers during the 1990s.

## Methods

### Subjects and investigated tankers

Forty-three men with a mean age of 43 years (range 19–60) were investigated. Twenty-two of them, all deck crewmembers, performed benzene-exposure-associated work tasks. Of 22 smokers in total, 11 were deck crewmembers (although smoking was prohibited while on deck, smoking did occur elsewhere in close connection to or during a work shift). Work was performed on four product and one chemical tanker during the summer months of 1995 and 1998. Ports visited were in Scandinavia and the UK. All tankers flied the Swedish flag. The tankers in the study had closed systems for loading and unloading but connection/disconnection of cargo lines, gauging, stripping, and tank cleaning were done manually. The cargo consisted of 95 or 98 octane leaded or unleaded gasoline, diesel products, Jet A or gas oil. However, exposure measurements and biomonitoring were only performed during transport of gasoline (95 or 98 octane, mostly unleaded). The benzene content of the cargo was not measured, but at the time would typically correspond to 3–4% [Jarvholm *et al.*, 1996; Conservation of Clean Air and Water in Europe (CONCAWE), 1998].

The working schedule for each seafarer was 4-h work followed by 8-h rest. The following 12 h involved a work shift with navigation or maintenance work away from the tanks (non-exposure). Work on deck on the cargo tanks (loading/unloading; maintenance) was defined as benzene-exposed work, else the monitored worker was defined as non-exposed. The deck crewmembers did not use any respiratory protection during a work shift. The number of work shifts included in the study was 39 work shifts for exposed workers, and 35 work shifts for non-exposed. Each monitoring session on board a tanker lasted ~ 1 week. Information on occupational factors (e.g. work tasks, on/off duty) outside this time frame for any individual seafarer was not collected.

The study was approved by the Ethical Board of Gothenburg (D-nr 170-93) and carried out after informed consent by the crewmembers.

### Sampling and chemical analysis

#### Sampling

Benzene in the breathing zone was collected with a personal passive dosimeter worn during a complete 4-h work shift (4-h time-weighted average [TWA]) among the exposed. For unexposed, only a couple of measurements were made to ascertain the background exposure. Samples for benzene in end-expiratory air (AlvBe) together with urinary samples for unmetabolized benzene (UBe) and ttMA were taken minutes prior to a work shift and at the end of that shift in a non-exposed part of the ship. An occupational hygienist and a nurse followed each ship for observation of the sampling schedule. The number of samples for all work shifts are given in Table 1. Sample numbers differed mostly out of practical reasons, e.g. lack of time or no urge to urinate. Number of analyses differed due to mishaps at the laboratory.

**Table 1.** Number of work shifts with and without benzene exposure and number of samples expressed in percentage (%) available for analysis.

	Unit	Work shifts with exposure (*N* = 39)		Work shifts without exposure (*N* = 35)	
		N	%	N	%
ExpBe	mg m^−3^	38	97	12	34
AlvBe_pre	ng L^−1^	18	46	1	3
AlvBe_post	ng L^−1^	21	54	0	0
UBe_pre	ng L^−1^	37	95	32	91
UBe_post	ng L^−1^	36	92	25	71
ttMA_pre	µg L^−1^	27	69	27	77
ttMA_post	µg L^−1^	27	69	27	77

#### Air monitoring

A diffusion sampler with an active charcoal sorbent (SKC 575-001, SKC Inc., Eighty-Four, PA, USA) was used throughout the work shift. Benzene was subsequently desorbed by carbon disulphide and analysed by gas chromatography with flame ionization detector (FID) according to standard technique [National Institute for Occupational Safety and Health (NIOSH), 1984]. The limit of detection (LOD) for a 4-h measurement was 0.02 mg m^−3^. Complementary continuous recording of total hydrocarbons was carried out during a couple of work tasks, using a photoionization detector (MTIP, Photovac 2200, Photovac Inc., Waltham, MA, USA).

#### Benzene in alveolar air

The techniques used for alveolar air sampling and analysis have been described in detail elsewhere (Ljungkvist and Nordlinder, 1995). In short, the sampling device consisted of a modified peak expiratory flow (PEF) meter, through which the subject exhaled by an exchangeable paper mouthpiece, with the air passing a one-way valve and out through a plastic tube. The sampling tube was inserted between the valve and the plastic tube, and connected to a syringe-type manual pump (Kitagawa AP-1, Komoy Co, Kawasaki, Japan). Alveolar air was sampled by retracting 100 ml of air with the manual pump during the last part of exhalation, into a glass tube with the Tenax TA adsorbent for benzene. The glass tube was then plugged until analysis in the laboratory, where benzene was thermally desorbed for analysis by gas chromatography and FID detection. The LOD was 0.5 ng L^−1^ for a 100 ml breath sample.

#### Biomarkers in urine

Urine samples were collected in 250 ml polyethylene bottles. Aliquots were immediately transferred to different containers according to the different analysis specifications before storage at about −20°C.

Samples for the determination of unmetabolized benzene in urine (UBe) were kept in 125 ml glass bottles with Teflon caps. An aliquot of 50 ml was transferred to a device for dynamic headspace, where the purging gas passed an adsorbent tube filled with Tenax TA. The tube was subsequently thermally desorbed and analysed using two-dimensional chromatography and FID detection. The LOD for the method was 7 ng L^−1^ (Ljungkvist *et al.*, 2001).

ttMA was analyzed with an in-house development of a method presented by Ducos *et al.* (1992). The analyte was concentrated on a strong anion exchange column and subsequently analyzed by two-dimensional reversed phase liquid chromatography and UV-detection. The LOD was 1 µg L^−1^.

### Statistical analysis

Statistical calculations were done in SAS 9.4. Data were assessed for normality by use of Shapiro–Wilks (significance level 0.05), skewness and kurtosis and visual inspections of q-q plots. A lognormal distribution was found to best describe the random effects. Results are given with the antilog of data. For measurements below LOD, half the LOD was used in the calculations (three benzene exposure measurements among the non-exposed). No data were defined as an outlier.

The difference between pre- and post-shift samples was analysed by paired *T*-test. For comparisons between groups, the *T*-test procedure was used. The geometric mean with a 95% confidence interval was derived with the proc univariate data/cibasic in SAS. If the number of samples were below three, no significance testing was made. Linear regression analysis for correlation was done with Pearson on the logarithmic values. Multiple regressions included age and smoking habits (the general linear model procedure).

## Results

### Benzene in air

The geometric mean of benzene exposure during a 4-h work shift was 0.45 (range 0.02–143) mg m^−3^ for exposed, and 0.02 (0.01–0.15) mg m^−3^ for non-exposed. The difference between exposed and non-exposed was significant (*P* < 0.0001) (Table 2). Five work shifts (three with tank cleaning, one with loading, and one including stripping) exceeded the Swedish OEL of 1.5 mg m^−3^. The highest exposure was noted for a tank cleaning operation (143 mg m^−3^).

**Table 2.** Number of samples (*N*), arithmetic mean (AM) with minimum and maximum values, and geometric mean (GM) with 95% confidence intervals (95% CIs) for benzene exposure measurements (4-h TWA; mg m^−3^).

		*N*	AM	Range (min–max)	GM	95% CI	*P* value^*a*^
Non-exposed		12	0.04	0.01–0.15	0.02	0.01–0.04	
Exposed		38	4.98	0.02–143	0.45	0.25–0.83	<0.0001
	Loading^*b*^	18	0.58	0.02–7.10	0.17	0.10–0.34	<0.0001
	Unloading^*b*^	12	0.62	0.06–1.90	0.35	0.17–0.74	<0.0001
	Stripping^*c*^	2	3.70	1.00–6.40	2.53		
	Tank cleaning^*d*^	6	27.34	1.90–143	6.62	1.23–35.49	<0.0001

^*a*^Significant difference from non-exposed.

^*b*^Closed handling.

^*c*^Manual, open tanks.

^*d*^Manual.

### Benzene in breath and benzene biomarkers in urine

The geometric mean of AlvBe among exposed increased significantly from 19 in pre-shift to 90 ng L^−1^ in post-shift (*n* = 18; *P* = 0.0018). The corresponding increases for UBe was 179–541 ng L^−1^ (*n* = 35; *P* < 0.0001) and for ttMA 129–408 µg L^−1^ (*n* = 27; *P* < 0.0001). Tables 3 show pre- and post-shift values for AlvBe, UBe, and ttMA in exposed and non-exposed.

**Table 3.** Number of samples (*N*), arithmetic mean (AM), range (min–max), and geometric mean (GM) with 95% confidence intervals (95% CIs) and *P* value for pre- and post-shift values of benzene in alveolar air (AlvBe), benzene in urine, and ttMA in urine (UBe and ttMA, respectively).

		Exposed				Non-exposed			
	Unit	*N*	AM (range)	GM (95% CI)	*P* value	*N*	AM (range)	GM (95% CI)	*P* value
AlvBe_pre	ng L^−1^ breath	18	25.4 (4.5–86.0)	19.2 (13.2–27.9)		1			
AlvBe_post	ng L^−1^ breath	21	538.0 (10.1–6800)	89.7 (41.0–196)	*P* = 0.0018				No data
UBe_pre	ng L^−1^ urine	37	893.4 (13.0–20 393)	179.1 (108.4–296)		32	311.5 (8.9–1854)	115.9 (68.0–197.4)	
UBe_post	ng L^−1^ urine	36	2771.0 (31.0–42 607)	541.2 (315.1–929.5)	*P* < 0.0001	25	273.1 (24.0–1195)	157.2 (99.8–247.6)	*P* = 0.0156
ttMA_pre	µg L^−1^ urine	27	181.7 (10.0–701)	128.6 (89.8–184.1)		27	353.8 (41.4–3630)	196.4 (136.1–283.3)	
ttMA_post	µg L^−1^ urine	27	755.9 (10.0–5099)	407.6 (251.6–660.4)	*P* < 0.0001	27	912.8 (42.0–14 400)	256.5 (155.5–423.0)	*P* = 0.2027

Among non-exposed, no analysis for AlvBe was possible (only one sample, pre-shift, with a value of 2.10 ng mL^−1^). The geometric mean for UBe in pre-shift increased from 115.9 to 157.2 ng L^−1^ in post-shift samples (*n* = 25; *P* = 0.0156) among non-exposed. Pre-shift ttMA among non-exposed was 196.4 compared to 256.5 µg L^−1^ in the post-shift samples (*n* = 27; *P* = 0.2027).

Pre-shift UBe was significantly higher in smokers compared to non-smokers (geometric mean 268.9 and 89.1 ng L^−1^, respectively; *P* = 0.0013). When stratifying for exposure, this difference was only found among the non-exposed (*P* = 0.0059). There were no significant differences in pre-shift AlvBe or ttMA between smokers and non-smokers.

### Relations between benzene in air and biomarkers

**Figure 1.**
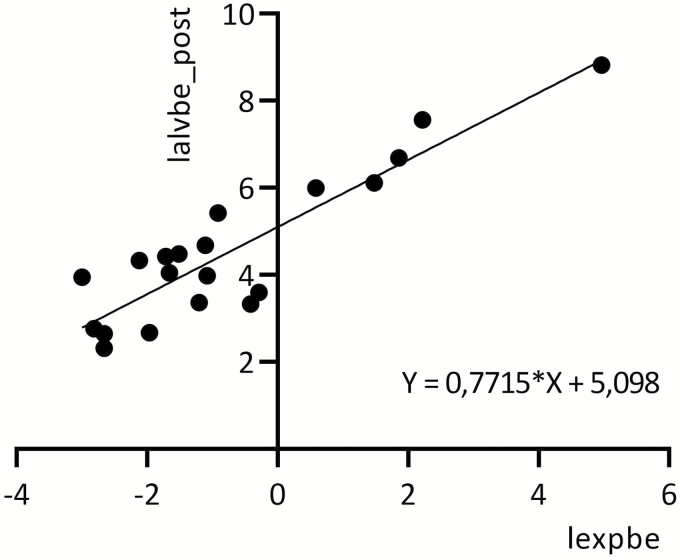
Relationship between logarithmic values of benzene in air (lexpbe) (4-h TWA) and post-shift benzene in alveolar air (lalvbe_post) for exposed tanker crewmembers.

Post-shift AlvBe correlated significantly with exposure to benzene in air (*r* = 0.89, *n* = 20, *P* < 0.0001) (Fig. 1).

**Figure 2.**
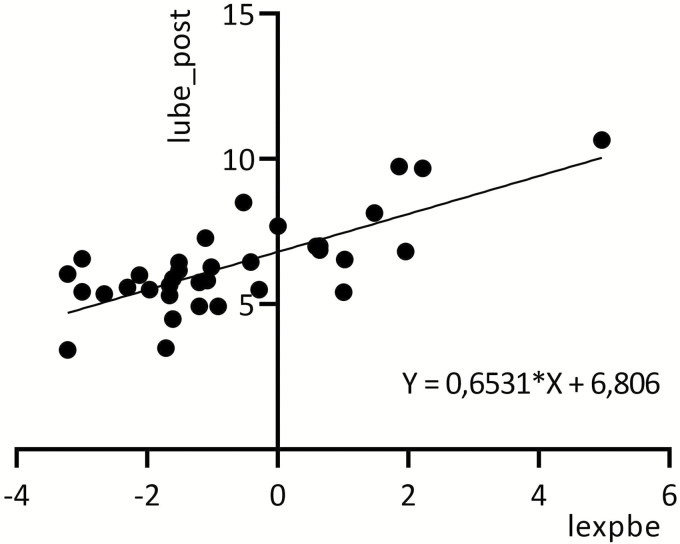
Relationship between logarithmic values of benzene in air (lexpbe) (4-h TWA) and post-shift benzene in urine (lube_post) for exposed tanker crewmembers.

**Figure 3.**
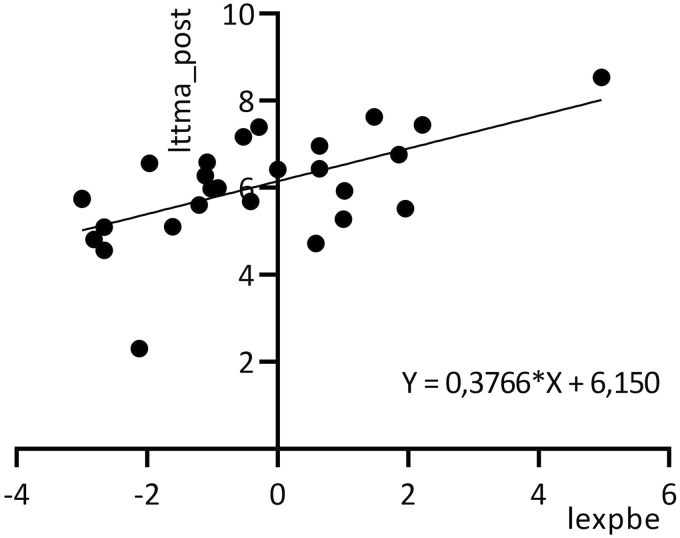
Relationship between logarithmic values of benzene in air (lexpbe) (4-h TWA) and post-shift ttMA (lttma_post) for exposed tanker crewmembers.

Benzene in air also correlated with post-shift UBe (*r* = 0.74, *n* = 35, *P* < 0.0001) and ttMA (*r* = 0.57, *n* = 27, *P* = 0.0011) (Figs 2 and 3).

A multiple regression analysis adjusting for smoker/non-smoker, cigarettes smoked during the work shift, and age did not change the level of significance between benzene in air and biomarkers among the exposed.

There was a good correlation between biomarkers among the exposed. Post-shift AlvBe correlated both with post-shift UBe (*r* = 0.84, *n* = 19, *P* < 0.0001) and post-shift ttMA (*r* = 0.49, *n* = 17, *P* = 0.027). Also, post-shift UBe correlated with ttMA (*r* = 0.54, *n* = 25, *P* = 0.0033).

## Discussion

Exposure to benzene for deck crewmembers on tankers handling gasoline during the mid-1990s was especially high during tank cleaning and stripping. The average exposure during a work shift expressed as the geometric mean was below the Swedish OEL, as well as the IMO-recommended OEL for marine shipping. However, it exceeded the new OEL-proposal by RAC (0.16 mg m^−3^, or 0.05 ppm) (RAC, 2018). Expressed in percentage of observations, 5% exceeded the Swedish OEL and 50% the RAC-proposal. Smoking did not correlate with biomarkers among exposed, presumably since benzene exposure from work on deck outweighed that from cigarette consumption. Such an interpretation on the effect of smoking on biomarkers at higher occupational benzene exposures was put forward in the recent ECHA proposal (ECHA, 2017).

A significant increase in UBe during a work shift among non-exposed indicated that this set of workers on tankers, not engaged in the handling of the cargo nor the tanks, may also be exposed to benzene. A couple of stationary exposure measurements on some tankers revealed benzene exposure in supposedly non-exposed areas on board (two of six readings from three different ships resulted in 0.13 and 0.15 mg m^−3^ of benzene in air, respectively; data not shown). Jacobs and co-authors in 2011 described similar results when measuring for benzene in air on a chemical tanker with mixed cargoes, detecting increased levels of benzene in areas of accommodation during cleaning and gas-freeing operations, although ventilation to accommodation was stopped (Jacobs *et al.*, 2011).

In the Methods section, we explained shortly reasons for any loss of data. This was especially problematic for the sampling of AlvBe, where roughly half the number of exposed workers could be monitored. The main reason for this was that the sampling had to be collected in an unexposed part of the tanker to avoid benzene contamination of the alveolar air samples, which turned out to be a bit cumbersome for the deck crewmembers.

Previous benzene exposure studies on tankers carrying gasoline have mainly been performed on older tanker types. No benzene exposure study for tankers carrying gasoline is reported after 1994. Of five studies identified, two reported exposure levels in 8-h TWA and the others for 5–45 min of sampling (Berlin, 1985; Christian and Eyres, 1986; Nordlinder and Ramnas, 1987; Bates *et al.*, 1994; Moen *et al.*, 1994). Our study was performed in the mid-1990s on product and chemical tankers with closed systems for loading and unloading, but they still involved manual work tasks for topping, stripping, and tank cleaning. These tankers are generally no more in service in the Western countries, where tankers now generally have fully automated systems. However, such tankers may still be operative in other parts of the world with less stringent legislation. In fact, the tankers in our study are now sailing the flags of Comoros, Panama, Tanzania, Nigeria, and Russia. In addition, with increased outsourcing in the trade, high benzene exposure may currently have shifted from deck crewmembers to workers in firms specialized in maintenance work, where knowledge on exposures are generally much less studied.

Peak exposures, generally considered for a maximum of 15-min exposure, has previously been summarized as quite low for marine deck crews in comparison with other occupations handling petroleum products, averaging 0.3 and 0.6 mg m^−3^ in the 90th percentile (ECHA, 2017). Our study revealed especially high exposures during stripping and tank cleaning (Table 2), and sometimes well beyond these levels. High exposures during tank cleaning operations have previously been described in the literature, although not specifically for gasoline transporting tankers (Williams *et al.*, 2005; Jacobs *et al.*, 2011). In particular, Kirkeleit and co-authors found a maximum individual benzene exposure of 54.6 mg m^−3^ (geometric mean 0.89 mg m^−3^; measuring for 43–538 min) during tank cleaning operations on a crude oil tanker (Kirkeleit *et al.*, 2006). The benzene content was not measured, but would correspond to around 0.52% according to the authors, which is considerably lower than for gasoline.

The biomarkers correlated well with benzene exposure in air. Our study indicated that all three biomarkers (benzene in alveolar air, benzene in urine, and ttMA in urine) might be used for exposure assessments on a group level for low occupational benzene exposure levels (low in the sense of beneath the current OEL). Since benzene in alveolar air has a very short half-elimination time (minutes), and the correlation was less strong for ttMA, benzene in urine seems the most appropriate biomarker. This conclusion is supported by the current recommendations of appropriate benzene biomarkers in low-exposure settings, that is benzene in urine or SPMA (ECHA, 2017; RAC, 2018).

It is especially the risk of leukaemia that generally is considered when establishing an OEL, usually considering a standard 40 years of exposure in extrapolation models. However, there are stated controversies regarding the use of such a model in benzene risk assessments, since biological effects (e.g. reduction in white blood cell counts, haematologic malignancy) have been associated with recent rather than long-term exposures, and with repeated peak exposures rather than with a cumulative exposure. The matter is discussed in the report by ECHA, which comments the fundamental use of the Pliofilm cohort in risk assessment of benzene exposures (see p. 97, third paragraph, in ECHA, 2017): (quote) ‘Finally, while the most commonly used exposure metric in the studies described was cumulative exposure in ppm-years, some studies have found indications that average exposure in ppms or number or level of peak exposures might play a role as well’ (ECHA, 2017). The subject is also discussed in other studies on the complex human metabolism of benzene in relation to patterns and intensity of benzene exposures (Kim *et al.*, 2006; Rappaport *et al.*, 2009; Vlaanderen *et al.*, 2011). We are currently investigating the odds ratios of leukaemia and other blood malignancies in a case-referent study on Swedish seafarers on product tankers in relation to the implementation of automation.

## Conclusions

Our study showed that measuring benzene in end-exhaled air and associated biomarkers was feasible under field conditions. Post-shift samples of unmetabolized benzene in urine seemed the most appropriate of the measured biomarkers for occupational benzene exposure assessment on a group level.

Deck crewmembers operating on tankers transporting gasoline with closed systems but still manual operations had an average benzene exposure under the current IMO OEL of 3.25 mg m^−3^ (1 ppm) but above the proposed new OEL within EU of 0.16 mg m^−3^ (0.05 ppm). Manual work tasks may lead to high benzene exposure, well above the Swedish STEL. Results suggested benzene exposure even for seafarers not directly involved in handling of the cargo.

## Funding

This work was funded by the Swedish Council for Work Life Research and The Assar Gabrielssons Foundation.
